# A global characterization of the translational and transcriptional programs induced by methionine restriction through ribosome profiling and RNA-seq

**DOI:** 10.1186/s12864-017-3483-2

**Published:** 2017-02-17

**Authors:** Ke Zou, Qi Ouyang, Hao Li, Jiashun Zheng

**Affiliations:** 1 The State Key Laboratory for Artificial Microstructures and Mesoscopic Physics, School of Physics, Peking University, Beijing, 100871 China; 2 Department of Biochemistry and Biophysics, University of California, San Francisco, CA 94158 USA; 3 Peking-Tsinghua Center for Life Sciences and Center for Quantitative Biology, Academy for Advanced Interdisciplinary Studies, Peking University, Beijing, 100871 China

## Abstract

**Background:**

Among twenty amino acids, methionine has a special role as it is coded by the translation initiation codon and methionyl-tRNAi (Met-tRNAi) is required for the assembly of the translation initiation complex. Thus methionine may play a special role in global gene regulation. Methionine has also been known to play important roles in cell growth, development, cancer, and aging. In this work, we characterize the translational and transcriptional programs induced by methionine restriction (MetR) and investigate the potential mechanisms through which methionine regulates gene expression, using the budding yeast *S. cerevisiae* as the model organism.

**Results:**

Using ribosomal profiling and RNA-seq, we observed a broad spectrum of gene expression changes in response to MetR and identified hundreds of genes whose transcript level and/or translational efficiency changed significantly. These genes show clear functional themes, suggesting that cell slows down its growth and cell cycle progression and increases its stress resistance and maintenance in response to MetR. Interestingly, under MetR cell also decreases glycolysis and increases respiration, and increased respiration was linked to lifespan extension caused by caloric restriction. Analysis of genes whose translational efficiency changed significantly under MetR revealed different modes of translational regulation: 1) Ribosome loading patterns in the 5′UTR and coding regions of genes with increased translational efficiency suggested mechanisms both similar and different from that for the translational regulation of Gcn4 under general amino acid starvation condition; 2) Genes with decreased translational efficiency showed strong enrichment of lysine, glutamine, and glutamate codons, supporting the model that methionine can regulate translation by controlling tRNA thiolation.

**Conclusions:**

MetR induced a broad spectrum of gene expression changes at both the transcriptional and translational levels, with clear functional themes indicative of the physiological state of the cell under MetR. Different modes of translational regulation were induced by MetR, including the regulation of the ribosome loading at 5′UTR and regulation by tRNA thiolation. Since MetR extends the lifespan of many species, the list of genes we identified in this study can be good candidates for studying the mechanisms of lifespan extension.

**Electronic supplementary material:**

The online version of this article (doi:10.1186/s12864-017-3483-2) contains supplementary material, which is available to authorized users.

## Background

Methionine is one of two sulfur-containing amino acids that are incorporated into proteins during translation. Among twenty amino acids, methionine plays a special role in the biosynthesis of proteins because its codon AUG is also the most common translation initiation codon. In eukaryotes, the binding of the anticodon of the initiator Met-tRNA to the initiation codon AUG is required for initiating translation [1]. This interaction is highly conserved across species. Met-tRNA is required for the assembly of 40S ribosome and thus may regulate the mechanism of ribosome scanning and entry, potentially serving as an important control point for translation [1–3]. Since translational regulation is a key step in gene regulation, sensing intracellular methionine level and adjusting the global gene expression program through translational control may be an important strategy to coordinate cell’s metabolic state with its growth.

Methionine has also been known to play important roles in a wild range of biological phenomena including growth, development, fertility, cancer and aging [4–9]. It has been widely reported that methionine intervention can effectively regulate the lifespan of numerous model organisms. In particular, methionine restriction (MetR) has been shown to extend the lifespan of a range of species, including yeast, worm, fly and mouse [10–13]. It has also been suggested that the lifespan extension by caloric restriction, defined as reduced caloric intake without malnutrition, can be attributed to methionine restriction [6, 14]. In addition to the effect on lifespan, methionine restriction also slows or reduces many characteristics associated with senescence, such as immune and lens aging, increased IGF-I and insulin levels, and cumulated oxidative damages [15, 16]. Methionine restriction has also been studied extensively in anticancer therapies, either alone or in association with the other treatments, and is considered as a useful therapeutic strategy for treating various cancers [17, 18]. Thus, characterizing the global gene expression program induced by MetR and understanding the mechanisms by which MetR regulates gene expression are important not only for understanding the basic principles of gene regulation but also for promoting human health.

Translational regulation by general amino acid starvation has been extensively studied and the pathway involved has been elucidated before [19, 20]. In the canonical model, amino acid starvation leads to the accumulation of uncharged tRNA, activating the Gcn2 kinase, which phosphorylates eIF2 (the Eukaryotic Initiation Factor 2), ultimately affecting the translation [21, 22]. As a general strategy for sensing amino acid depletion, this may also be the mechanism to sense and respond to MetR. Methionine may also work through other mechanisms to affect translation. It has been reported that intracellular methionine availability can regulate cellular translational capacity and metabolic homeostasis by controlling the thiolation status of the wobble-uridine (U34) nucleotides on lysine, glutamine, or glutamate tRNAs [23]. Methionine may also affect gene expression by converting to S-adenosyl methionine [24], which serves as the predominant methyl donor for rRNA-tRNA modifications and DNA/protein methylations. Although there has been significant progress in understanding the roles methionine may play in gene regulation, a systematic study on the global gene expression program controlled by methionine, especially at the translational level, is still lacking.

In this work, we use ribosomal profiling and RNA-seq to compare the translational and transcriptional profiles of cells growing in the normal and methionine restricted media. We systematically characterize the translational and transcriptional programs induced by methionine restriction and investigate the potential mechanisms through which methionine regulates gene expression, using the budding yeast *S. cerevisiae* as the model organism.

## Methods

### Yeast strains and media

Yeast strains used for the ribosomal profiling and RNA-seq experiments were BY4741 (MATa his3Δ1 leu2Δ0 met15Δ0 ura3Δ0).

Synthetic Dextrose (SD) medium and methionine restriction (MetR) medium was used in the ribosome profiling/RNA-seq experiments, The SD medium contained 2% (wt/vol) glucose, 6.7 g/L yeast nitrogen base (YNB) without amino acid, 20 mg/L Adenine, 20 mg/L L-Arginine HCL, 100 mg/L L-Aspartic Acid, 20 mg/L L-Histidine HCL, 100 mg/L L-Leucine, 30 mg/L L-Isoleucine, 30 mg/L L-Lysine HCL, 20 mg/L L-Methionine, 50 mg/L L-Phenylalanine, 200 mg/L L-Threonine, 20 mg/L L-Tryptophan, 30 mg/L L-Tyrosine, 20 mg/L Uracil, 150 mg/L L-Valine, 100 mg/L glutamic acid and 4 g/L serine. The MetR media has the similar ingredients as the SD medium except for 4 mg/L L-Methionine concentration. Media were freshly made before the experiments. All nutrients were purchased from Sigma-Aldrich Corporation.

### Ribosome profiling and RNA-seq of cells growing in SD vs. MetR media

The initial cell culture was incubated in 300 ml SD medium overnight to an OD600 0.8 ~ 1.0, then diluted by five fold using fresh SD media and incubated for another 4 h under 30 °C to an OD600 0.8 ~ 1.0. The sample was then divided equally into two aliquots. Cells were separated from the media by spin-down at 3000 g for 5 min and re-suspended in SD and MetR media respectively. All samples were incubated for another hour before harvesting. All the steps were carried out at 30 °C.

Ribosomal profiling and RNA-seq experiments were carried out using the protocol developed by Ingolia et al. [25] Raw sequences were obtained from Illumina Hiseq 2000.

### Sequence analysis and quantification of differential gene expression

Sequence reads were aligned to the most recent S. cerevisiae genome using SOAPaligner/SOAP2 (2.21) with default setting [26]. After trimming off the adapters, reads aligned to rRNA and tRNA sequences were filtered out. The rest of the reads were then aligned to the genome sequence. Finally, reads that did not align to the genome were aligned to all the CDSs to retain those covering the splicing junctions. After the alignment, we counted the number of reads starting at each position across the whole genome; for ribosomal footprinting data, the starting position of each read was shifted by 15 bps towards the 3′ end, to adjust for the offset due to ribosome protection. To get the abundance of reads covering each gene, we sum all reads with starting position from the start to the stop codon, excluding the first 50 bps from the transcription start site (TSS), to alleviate the effect of the biased distribution of reads around the TSS [25, 27].

For a reliable gene expression comparison between two conditions, we excluded genes with less than 128 total raw reads (combining the reads from the two conditions) [27]. We computed the fold change of mRNA or footprint as the ratio of the corresponding reads from the two conditions, with total reads normalized to adjust the median fold change to 1. To estimate the statistical significance of the fold change, we did the following analysis: for each gene *g*_*i*_, we collected the other 100 genes with the most similar number of reads and compute the standard deviation *σ*_*i*_ from the log (fold_change) of these 101 genes. We then computed the ${z}_i=\frac{ \log \left({F}_i\right)}{\sigma_i}$, where *F*_*i*_ is the fold change of *g*_*i*_ comparing MetR and SD. Then a *p*-value was calculated from the *z*_*i*_ to measure the significance of the fold change.

### Quantification of translational efficiency changes

The translational efficiency changes were calculated as the ratio of ribosomal footprints fold change to mRNA fold changes for each gene. Translational efficiency change Z-score is calculated by normalized the efficiency change with the standard deviation.

### Calculation of the TF module z-scores and KEGG pathway z-scores

We used the transcription factor targets from the analysis by McIssac et al. based on the systematic ChIP-chip data [28], using 0.001 as the *p*-value cutoff and the strongest conservation between species [29]. We computed the rank sum test z-scores comparing the fold changes of the target genes vs. none-target genes for each transcription factor. The sign of the z-score reflects the overall direction of the gene expression change in the modules; positive z-score indicates overall induction and negative z-score indicates overall repression. We used TF modules with at least 15 targets for this analysis. We used a similar method to compute the KEGG [30] pathway z-scores by grouping the genes from the same KEGG pathway.

### Flow cytometer measurement of the protein abundance changes upon methionine restriction

Yeast GFP-tag strains were selected from the yeast GFP library [31]. For each GFP strain, we picked three single clones from the plate and cultured them overnight to saturation. We then diluted each cell culture on the second day and grew them to OD600 0.1–0.3 in a 96 well plate. Cells were collected (by spinning down at 3000 × g for 5 mins and removing the supernatant) and re-suspended in MetR or SD media. We then used flow cytometer to measure the GFP signals (FITC channel) in each sample after 4 h (for about 50,000 cells per sample). The cellular GFP concentration was computed by normalizing the GFP signal with the cell size using Forward Scattering Signal (FST channel) for each individual cell. The difference of GFP concentration between MetR and SD was computed as the ratio of the medians of the normalized GFP under the two conditions. Then the mean GFP fold change was calculated from the three biological replicates.

## Results

### Ribosomal profiling and RNA-seq revealed a broad spectrum of transcriptional and translational changes induced by MetR

**Fig. 1:**
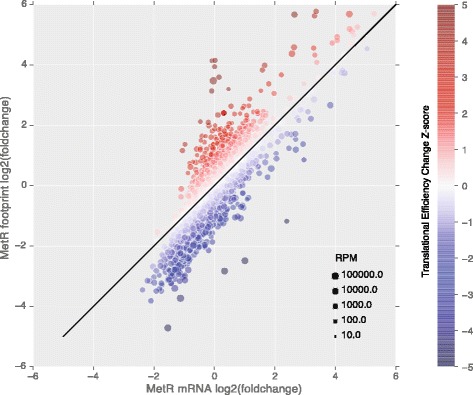
RNA-seq and ribosomal profiling revealed global transcriptional and translational regulation by methionine restriction. Log2 of the fold change (MetR vs. SD) of ribosomal-protected RNA is plotted against Log2 fold change of the mRNA, for all the genes. The size of the dots represents the number of reads (per one million total reads) for the gene. Translational efficiency is measured by the ratio of ribosomal footprints fold-changes to the mRNA fold-changes, quantified by a z-score (indicated by the color, see Methods)

We performed global gene expression profiling by RNA-seq and ribosomal profiling [27, 32], comparing cells growing in normal vs. MetR (0.2 times the methionine concentration in the normal media) conditions. The sequencing result is of high quality with at least 50× coverage per sample (summary statistics of the sequencing reads shown in Additional file 1). Ribosome profiling quantifies ribosome protected RNA (footprint) for all the genes in the genome and thus can measure the translational efficiency when combined with the total amount RNA from the RNA-seq data. We observed a broad spectrum of gene expression changes in response to MetR, both at the transcriptional and the translational levels (Fig. 1, Additional file 2 and Additional file 3: Figure S1). For the majority of the genes that changed expression, the regulation is at the transcriptional level, as the fold change of the footprint is proportional to the fold change of the transcript level (i.e., the majority of the points fall on the diagonal). There is a subset of genes whose translational efficiency (defined as the ratio of the ribosomal footprints to the total RNA) are increased or decreased compared to all the genes (Fig. 1, dots with dark red or blue color), indicating that they are under translational regulation. We observed that a subset of genes with decreased transcriptional level tend to have a decreased translational efficiency, suggesting that they are under both transcriptional and translational control (Fig. 1, blue dots). Overall there are 110/149 genes whose footprints went up/down by more than four-fold under MetR, and 149/232 genes increased/decreased their translational efficiency by more than two-fold.

We found that genes with increased expression (footprint_fold_change > 4) are enriched for those involved in the amino acid biosynthetic process, including genes coding for enzymes for methionine biosynthetic pathway and sulfate assimilation. Those down-regulated genes (footprint_fold_change < ¼) are enriched for protein synthesis (ribosomal genes) and RNA methylation (Additional file 4). We have selected a few top repressed/induced genes and measured the corresponding protein level change upon methionine restriction using flow cytometry and GFP reporter strains. The results are consistent with the footprint measurements (Additional file 5: Figure S2.)

**Fig. 2:**
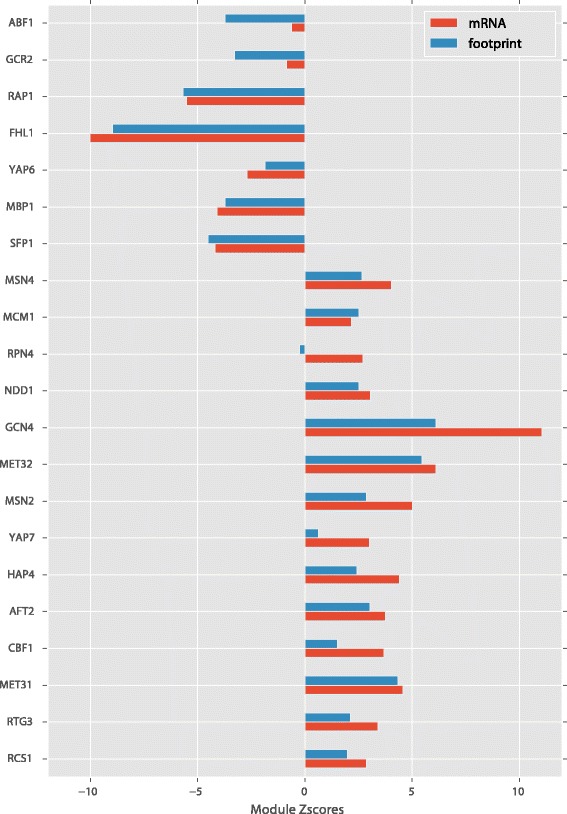
Transcription factors that play important roles in the regulation of gene expression by methionine restriction, identified by the transcription module analysis. Module Z-scores measures the collective change of all the targets of the transcription factor relative to other genes (see Methods). Transcription modules with z-scores > 2.5 or < −2.5 based on mRNA changes or footprint changes were shown

To identify the functional themes of the gene expression program, we analyzed the gene expression changes by organizing genes into functional groups with shared transcriptional regulators. Genes were grouped into transcription modules -- genes co-regulated by the same transcription factor, using the TF – target relationships previously identified by a genomic ChIP-chip analysis [29]. We then analyzed the expression change of genes in the TF modules collectively by calculating a z-score for the whole module (see Methods). This approach allows a simpler functional organization of the transcriptome and improves the statistical power when the targets of a TF have small but coherent fold changes. The analysis revealed that a number of TF modules are significantly up/down regulated (14 TF modules with z_score > 2.5, and 7 TF modules with z_score < −2.5 when using either the transcript level or the footprint level; Fig. 2, Additional file 6), with clear functional themes. TF modules down-regulated are involved in protein synthesis (ribosomal gene regulators RAP1, FHL1, SFP1), cell cycle progression (MBP1 for G1/S transition and ABF1 for DNA replication) and glycolysis (GCR2). TF modules up-regulated are involved in methionine biosynthesis (MET31, MET32, CBF1) and general amino acid starvation response (GCN4), general stress response (MSN2, MSN4), cellular maintenance (RPN4 for proteasome, RTG3 for mitophagy), respiration (HAP4), and iron utilization (RCS1, AFT2). These observations suggest that in response to MetR, cell slows down its growth and cell cycle progression and increases its stress resistance and cellular maintenance, in addition to the obvious increase of methionine pathway genes.

Interestingly, under MetR cell also decreases glycolysis and increases respiration, and increased respiration was linked to lifespan extension caused by caloric restriction, suggesting that MetR may also require increased respiration to extend lifespan [33].

We also performed a similar analysis using KEGG [30] pathways. Overall the results are consistent with the GO and TF module analyses. The pathway analysis also reveals a few interesting pathways missed by the GO analysis, including the induction of autophagy and ubiquitin-mediated proteolysis (for the full results, see Additional file 6), suggesting that the cell tries to recycle amino acids under Methionine restriction.

Because of methionine’s special role in regulating translation, we are particularly interested in the subset of genes that were subjected to translational regulation by MetR. No enrichment of gene ontology categories was found in genes that increased their translational efficiency more than two-fold. Genes with decreased translational efficiency are enriched for carboxylic acid metabolic process, urea metabolic process, amino acid biosynthetic pathway (except Methionine biosynthetic pathway) and protein synthesis (ribosomal genes) (Additional files 4 and 6). Ribosomal genes are suppressed at the mRNA level and suppressed even further at the footprint level. Similar results were also obtained from KEGG pathway analysis (Additional file 6).

### Potential mechanisms for translational regulation by MetR

**Fig. 3:**
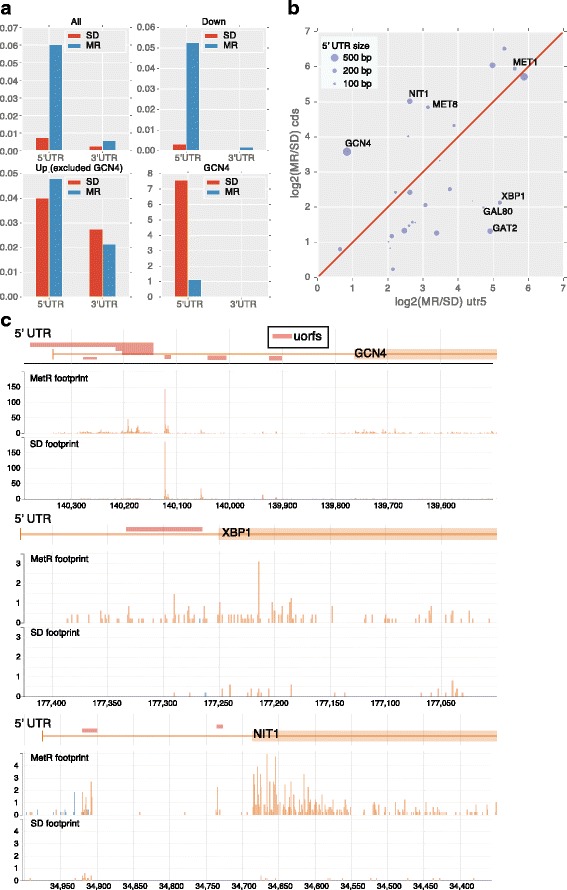
Ribosomal occupancy pattern of the 5′ UTR and the coding regions for translationally regulated genes. **a**. The ratio of ribosomal footprint reads in 5′UTR to open reading frame under SD and MetR condition, genes were grouped by their translational efficiency changes: Up: efficiency change Z-score > 2; Down: efficiency change Z-score < −2 and other genes. **b**. Scatter plot showing the fold change of reads in 5′UTR vs. the fold change of reads in the open reading frame for the genes with efficiency change > 2, dot size indicating the length of the 5′UTR. **c**. Distribution of the ribosomal footprint reads on 5′UTR and coding regions of GCN4, XBP1, and NIT1

To investigate the mechanisms for translational regulation by MetR, we analyzed the potential regulatory role of 5′UTRs in the change of translational efficiency induced by MetR. We analyzed ribosome loading patterns by calculating the ratio of reads on the 5′ UTR or 3′ UTR over the coding region (Fig. 3a) for the group of genes with increased, decreased or no efficiency changes (defined by the efficiency z-score cutoff 2 and −2). The ratio of reads on 5′UTR vs. coding region increase dramatically for most of the genes except for those with increased translational efficiency (Fig. 3a). The ratio for genes with increased translational efficiency (efficiency z-score > 2, excluding GCN4) was high in the SD condition (0.04 vs. 0.008), and does not change much under MetR. The translation of GCN4 is known to be regulated by the 5′uorfs [27, 34] and its 5′ to coding ratio decreased drastically under MetR condition. This is consistent with the canonical model of GCN4 regulation [19, 20] and with the previous ribosome profiling experiments under general amino acid starvation condition [27].

To further analyze the change of ribosome loading pattern under MetR, we compared the fold change of the footprint in the 5′UTR and the coding regions for each gene whose translational efficiency increased under MetR (Fig. 3b, c). There are three classes of genes with distinct patterns of the change of 5′UTR reads vs. that of the coding sequences. Class one genes increase the footprints significantly more in their coding region compared to the 5′UTR region (Fig. 3b, dots above the diagonal line), indicating significantly more loading of ribosome at the canonical start site. This group includes GCN4 and several other genes such as NIT1, suggesting that they might be regulated in the similar fashion as GCN4. Class two genes increase the footprints more in their 5′UTR region than the coding region (dots below the diagonal line, one example is XBP1, shown in Fig. 3c), suggesting that the increased translational efficiency was due to ribosome loading at the non-canonical start in the 5′UTR region. Thus the mechanism can be quite distinct from that for the regulation of GCN4. Class 3 genes show uniform changes in the 5′UTR region and the coding region (dots close to the diagonal line). These results suggest that even for the genes with increased translational efficiency, there are potentially distinct regulatory mechanisms for different genes. There is no obvious correlation between the length of the 5′UTR region and the class (Fig. 3b size of the dots).

### Translational repression of ribosome biogenesis genes by MetR strongly correlated with the higher codon frequency in lysine, glutamine and glutamate suggesting translational regulation through tRNA thiolation

**Fig. 4:**
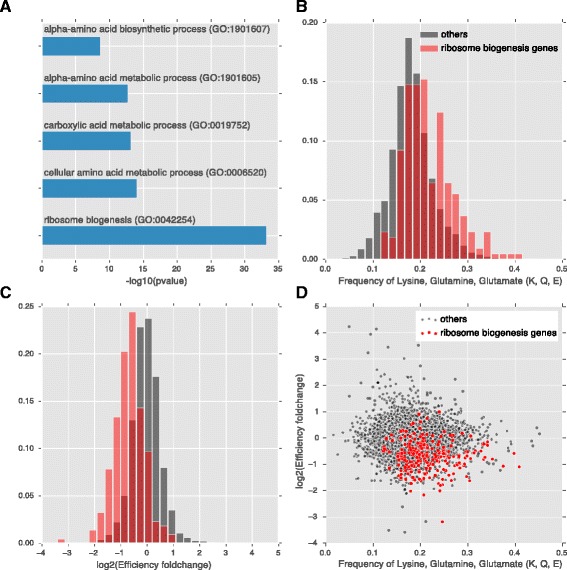
Correlation between translational efficiency and the codon frequency of lysine, glutamine and glutamate (K, Q, E) for the ribosome biogenesis genes. **a**. Top gene ontology categories enriched in genes with decreased translational efficiency (fold-change <1/2). **b**. Histogram of the codon frequency of KQE in ribosome biogenesis genes compared with other genes. **c**. Histogram of the translational efficiency fold-change comparing the ribosome biogenesis genes with other genes. **d**. Ribosome biogenesis genes showed a higher KQE frequency and decreased translational efficiency in the scatter plot

One potential mechanism through which methionine may directly regulate translation is through modulation of tRNA thiolation which is important for efficient translation of gene enriched in lysine (K), glutamine (Q) and glutamate (E) codons [23]. Under sulfur starvation, tRNA thiolation will be downregulated. If this mechanism operates under MetR condition, we expect that genes enriched with KQE codons will have lower translation efficiency. We calculated the Pearson correlation between the frequency of K, Q, E individually or combined with the translational efficiency changes under MetR (Additional file 7). There is a negative correlation (*r* = −0.037, *p* ~ 0.01) between the KQE frequency and translational efficiency. The negative correlation becomes stronger (*r* = −0.055, *p* ~ 0.0001) when considering only the frequency of K. This correlation becomes even more pronounced when considering specific gene categories. In the gene ontology analysis, we identified several groups of genes whose translational efficiency are significantly down-regulated by MetR, including the ribosome biogenesis genes (Fig. 4a, c). This group of genes also have a higher frequency of lysine, glutamine and glutamate codon (Fig. 4b), showing a significant negative correlation with the translational efficiency change (Fig. 4d). This suggested that the repression of the translational efficiency of ribosome biogenesis genes may be controlled by the thiolation pathway. For genes in other categories that are translational downregulated, there is no bias in the K, Q, E codon frequency. In addition, when excluding all the genes in the ribosome biogenesis category, there is no correlation between the codon frequency of KQE and the translational efficiency changes, indicating that the tRNA thiolation pathway can only explain part of the translational efficiency changes. There is no significant correlation between the translational efficiency changes and the frequency of methionine codon.

## Discussion

Translational regulation is a key step in gene regulation and plays an important role in cellular response to changing environment. So far translational regulation has been much less well studied compared to transcriptional regulation. The recent development of the ribosome profiling technique made it possible to study translational regulation at a fine resolution [25, 27]. As a special amino acid, methionine is coded by the translation initiation codon and methionyl tRNAi (Met-tRNAi) is required for the assembly of the translation initiation complex [19, 35], thus sensing the cellular level of methionine may be an important mechanism for controlling translation and for coordinating the metabolic state of a cell with its growth.

In this work, we have quantified the global transcriptional and the translational programs induced by methionine restriction, using ribosome profiling and RNA-seq. We have identified hundreds of genes whose transcript level and/or translational efficiency changed significantly. Analysis of transcriptional changes based on transcription modules revealed clear functional themes. While ribosomal genes and genes responsible for carbohydrate metabolism and cell cycle progression (in particular G1/S transition) are repressed, genes responsible for methionine and general amino acid synthesis, stress response, and cellular maintenance (e.g., regulated protein degradation and mitophagy) are induced, indicating that cell slows down its growth and increases its stress resistance and maintenance/repair in response to methionine depletion. Interestingly, MetR seems to induce respiration and decrease glycolysis, suggesting that the intra-cellular methionine level is coordinated with carbohydrate metabolism. Since methionine plays a key role in translational regulation, this suggests that regulation of translation is coordinated with metabolic state of the cell. It is also worth noticing that iron utilization is increased under MetR. Since methionine contains sulfur, this suggests that the cell’s response is to coordinate sulfur with iron, perhaps in making sulfur-iron clusters shown to be important for regulating lifespan [36].

Our analysis of genes whose translational efficiency is significantly changed by MetR suggested a few mechanisms for translational regulation through methionine. One well-studied mechanism for translational regulation is the regulation of Gcn4 under general amino acid starvation, which involves a pathway triggered by uncharged tRNA. Gcn4 translation is regulated by several upstream UORFs. Under normal condition, only the 5′ UORFs are translated. Under amino acid starvation condition, uncharged tRNA activates the Gcn2 kinase which phosphorylates the translation initiation factor EIF2-alpha, leading to the translation of Gcn4. Previous ribosomal profiling of general amino acid starvation showed high ribosome occupancy in the 5′UORF region of Gcn4 which significantly decreases upon AA starvation, and at the mean time AA starvation induces a drastic increase of ribosome occupancy in the coding region [27]. Consistent with this observation, we found a similar pattern of ribosome loading at Gcn4. In addition, we found several other genes with ribosome loading pattern similar to Gcn4, suggesting that they might be regulated in the similar fashion. Interestingly, we also found a group of genes with significantly increased translational efficiency, but with opposite ribosome loading patterns (Fig. 3b). These genes have much increased loading at their 5′UTR compared to their coding regions upon MetR, suggesting that the potential mechanism can be quite different from that for regulating Gcn4. Our study provided good candidate genes/reporters for the detailed mechanistic study of translational regulation.

**Fig. 5:**
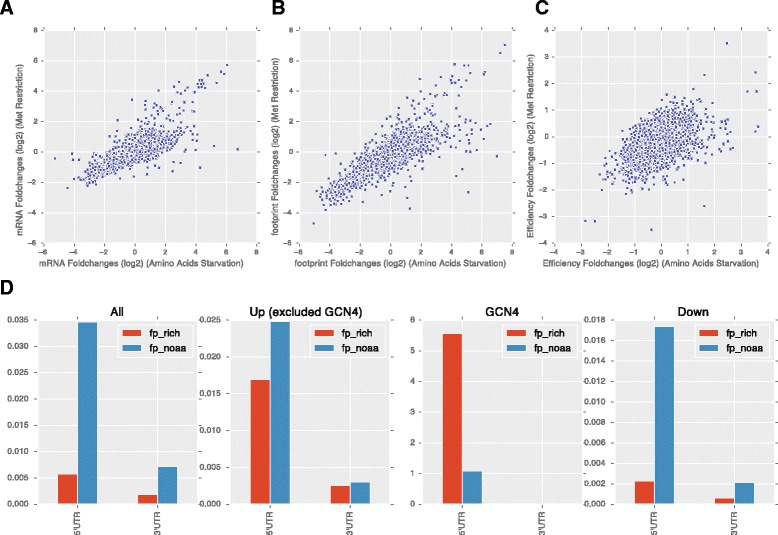
Comparison of the transcriptional and translational changes by general amino acid starvation and methionine restriction. Showing are scatter plots of the transcriptional changes (**a**), ribosomal footprint changes (**b**), translational efficiency changes (**c**), and ribosome occupancy of the 5′UTR and the coding regions under amino acid starvation condition compared with the rich media (**d**)

Previously, Laxman et al. suggested that methionine can regulate translation through modulation of tRNA thiolation. Their study indicated that the intracellular methionine level directly controls the thiolation status of wobbleuridine (U34) nucleotides present on lysine (K), glutamine (Q), or glutamate (E) tRNAs, and that thiolated tRNAs lead to more efficient translation of genes enriched for KQE codons [23]. Our analysis of genes whose translational efficiency significantly decreased under MetR lent additional support to this model. Using gene ontology (GO) analysis, we found the translational efficiency of rRNA processing genes are significantly downregulated by MetR, and that this group of genes is significantly enriched for KQE codons (Fig. 5). While Laxman et al. study employed analysis of the proteomes of thiolation mutants, our study directly measured the translational efficiency of all genes under MetR condition, providing complementary evidence supporting the thiolation model.

Translational regulation by general amino acid starvation has been studied previously by ribosome profiling [27]. We compare the gene expression profile of MetR and amino acid starvation [27]. Overall, there is significant overlap between the transcriptional and translational changes induced by MetR and amino acid starvation (Fig. 5a, b, Additional file 8: Figure S3), which is not surprising as methionine is also restricted in the amino acid starvation. Amino acid starvation induced a stronger translational efficiency change (Fig. 5c), while only a few genes show more efficiency change in MetR. The footprint read coverage changes in the 5′UTR region in amino acid starvation condition is similar to MetR, showing a strongly increased ratio of reads in 5′UTR over coding sequences for most of the genes. Genes with increased translational efficiency also start with a higher 5′UTR read ratio which increased only marginally under amino acid starvation (Fig. 5d) similar to MetR. Although these patterns are similar, the specificity of MetR allowed us to infer potential regulatory mechanisms directly related to methionine.

MetR is known to be able to extend the lifespan of a wide range of species. Our study identified a number genes with changed transcription and translational efficiency under MetR; these genes can be good candidates for analyzing the downstream effectors of lifespan extension by MetR. For example, increased autophagy and respiration have been linked to the lifespan extension by caloric restriction, which is another well-known regimen that extends lifespan across species. Future studies based on the genes we identified should provide new insight into the mechanism of lifespan extension by MetR.

## Conclusions

In this work, we characterize the translational and transcriptional programs induced by MetR and investigate the potential mechanisms through which methionine regulates gene expression, using the budding yeast S. cerevisiae as the model organism. Using ribosomal profiling and RNA-seq, we systematically compared the translational and transcriptional profiles of cells growing in the normal and methionine restricted media. We observed a broad spectrum of gene expression changes in response to MetR, including hundreds of genes whose transcript level and/or translational efficiency changed significantly. These genes fall into specific functional classes that are informative of the physiological state of the cell under MetR. Analysis of ribosome loading patterns of genes with increased translational efficiency suggested mechanisms both similar and different from the canonical model of translational regulation by general amino acid starvation. Analysis of the genes with decreased translational efficiency added support to the thiolation model of translational regulation by methionine. Since MetR extends the lifespan of many species, the list of genes we identified in this study can be good candidates for studying the downstream effectors of lifespan extension.

## Additional files

Additional file 1: Sequencing reads statistics. Reads statistics for the RNAseq and ribosome footprint (XLSX 27 kb)
Additional file 2: mRNA and footprint fold changes with *p*-values and translational efficiency under methionine restriction. There are 4 spread sheets in this file: 1. “MetR foldchanges”: Fold changes in mRNA and footprint. 2. “mRNA_foldchange with p-val”: Fold changes in mRNA with *p*-values. 3. “Footpring_foldchange with p-val”: Fold changes in footprint with *p*-values. 4. “MetR efficiency changes”: Translational efficiency changes under methionine restriction. (XLSX 2804 kb)
Additional file 3: Figure S1. Volcano plot of the fold change and *p*-values. (A). Transcription change under MetR and the associated *p*-value computed from mRNA data as described in the method, (B). Translation change under MetR and the associated *p*-value computed from footprint data. The *p*-values are provided in Additional file 2. (PDF 2491 kb)
Additional file 4: Gene ontology enrichment analysis of the genes with high footprint changes or translational efficiency changes. There are 4 spread sheets in this file: 1. MetR footprint UP 4 fold: Enriched GO biological process categories in the genes up-regulated under methionine restriction by more than four-fold in footprint level. 2. MetR footprint Down 4 fold: Enriched GO biological process categories in the genes down-regulated under methionine restriction by more than four-fold in footprint level. 3. MetR efficiency UP 2 fold: Enriched GO biological process categories in the genes with increased translational efficiency by at least two-fold. 4. MetR efficiency Down 2 fold: Enriched GO biological process categories in the genes with decreased translational efficiency by at least two-fold. (XLSX 20 kb)
Additional file 5: Figure S2. Validation of protein level changes under MetR by flow cytometer. Red bars are genes with increased footprint reads while blue bars represent genes with decreased footprint reads. The mean GFP fold changes and error bars are computed from three biological replicates. (PDF 160 kb)
Additional file 6: Module scores and KEGG pathway scores under Methionine Restriction and amino acids starvation. There are two spread sheets: 1. Module_scores: Transcription factor module scores and corresponding *p*-values from the fold change data under Methionine Restriction or amino acids starvation. 2. KEGG pathway_scores: KEGG pathway scores and corresponding *p*-values from the fold change data under Methionine Restriction or amino acids starvation. (XLSX 66 kb)
Additional file 7: Translational efficiency changes under Methionine Restriction and the codon frequency of Lysine(K), Glutamine(Q), Glutamic acid(E) and Methionine(M). This file shows the translational efficiency change for each gene and its codon frequency of Lysine(K), Glutamine(Q), Glutamic acid(E) and Methionine(M). In addition, the length of the gene and the 3′UTR and 5′UTR length are also listed. (XLSX 599 kb)
Additional file 8: Figure S3. Similar transcriptional and translational regulation under MetR and general amino acid starvation revealed by transcription factor modules: (A) module scores from mRNA data. (B) module scores from footprint data. (PDF 193 kb)

## Abbreviations

ChIP: Chromatin immunoprecipitation
KQE codons: Lysine (K), glutamine (Q) and glutamate (E) codons
MetR: Methionine restriction
SOAPaligner/soap2: A member of the Short Oligonucleotide Analysis Package
TSS: Transcription start site
