# Supplementary figures and images for: A global characterization of the translational and transcriptional programs induced by methionine restriction through ribosome profiling and RNA-seq
Source: BMC Genomics. 2017 Feb 17;18:189. doi: 10.1186/s12864-017-3483-2 (PMC5316152; doi:10.1186/s12864-017-3483-2)

**A**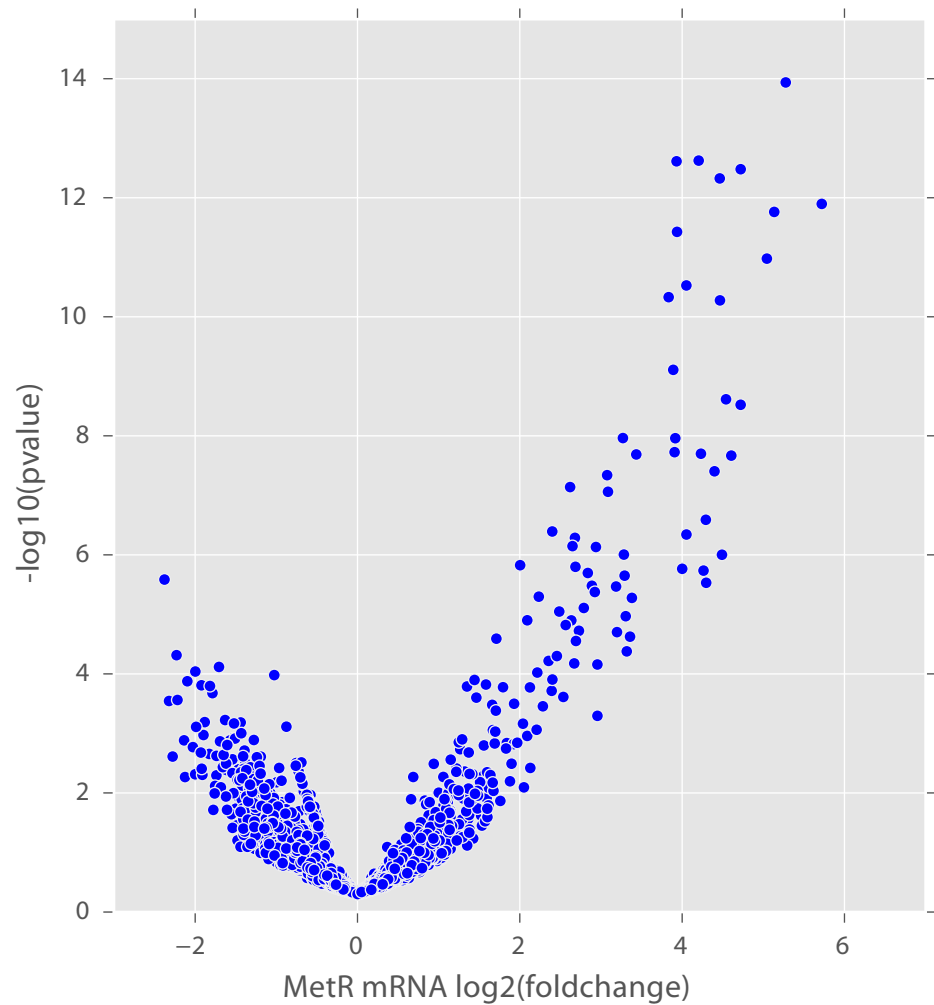**B**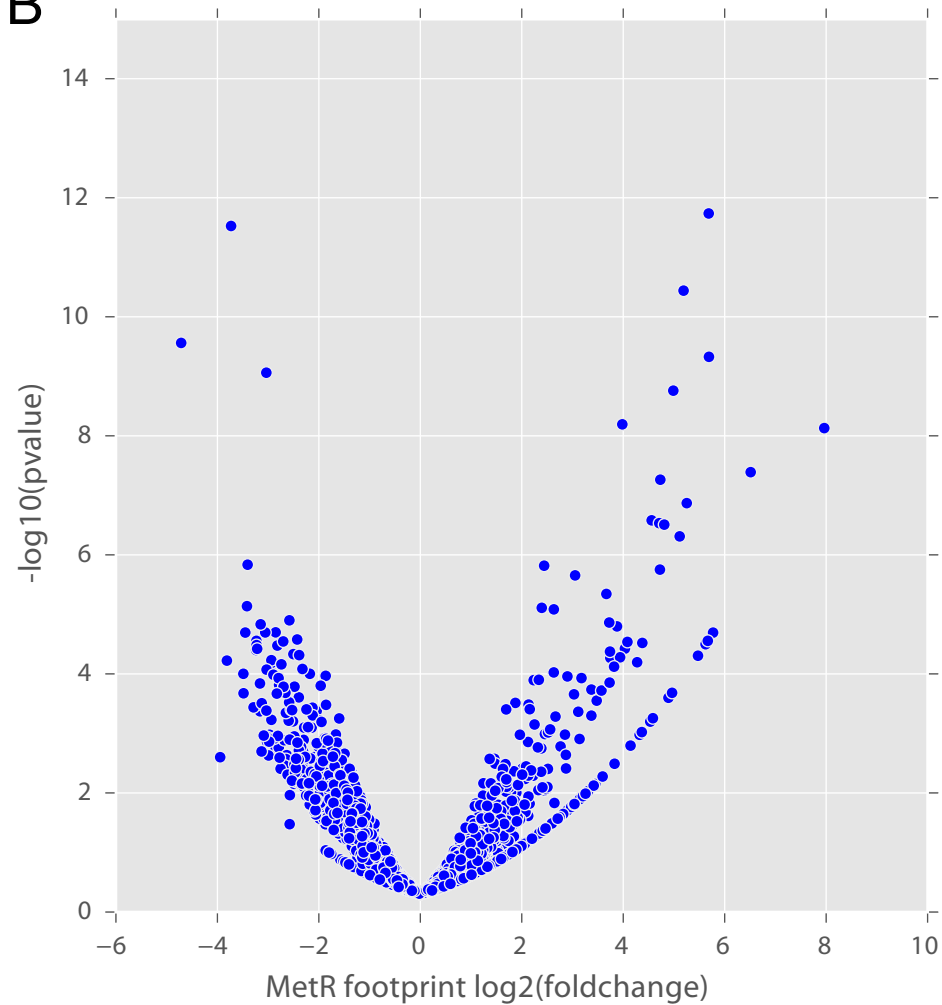

Supplement: Additional file 3: Figure S1. — Volcano plot of the fold change and p-values. (A). Transcription change under MetR and the associated p-value computed from mRNA data as described in the method, (B). Translation change under MetR and the associated p-value computed from footprint data. The p-values are provided in Additional file 2. (PDF 2491 kb) [file 12864_2017_3483_MOESM3_ESM.pdf]

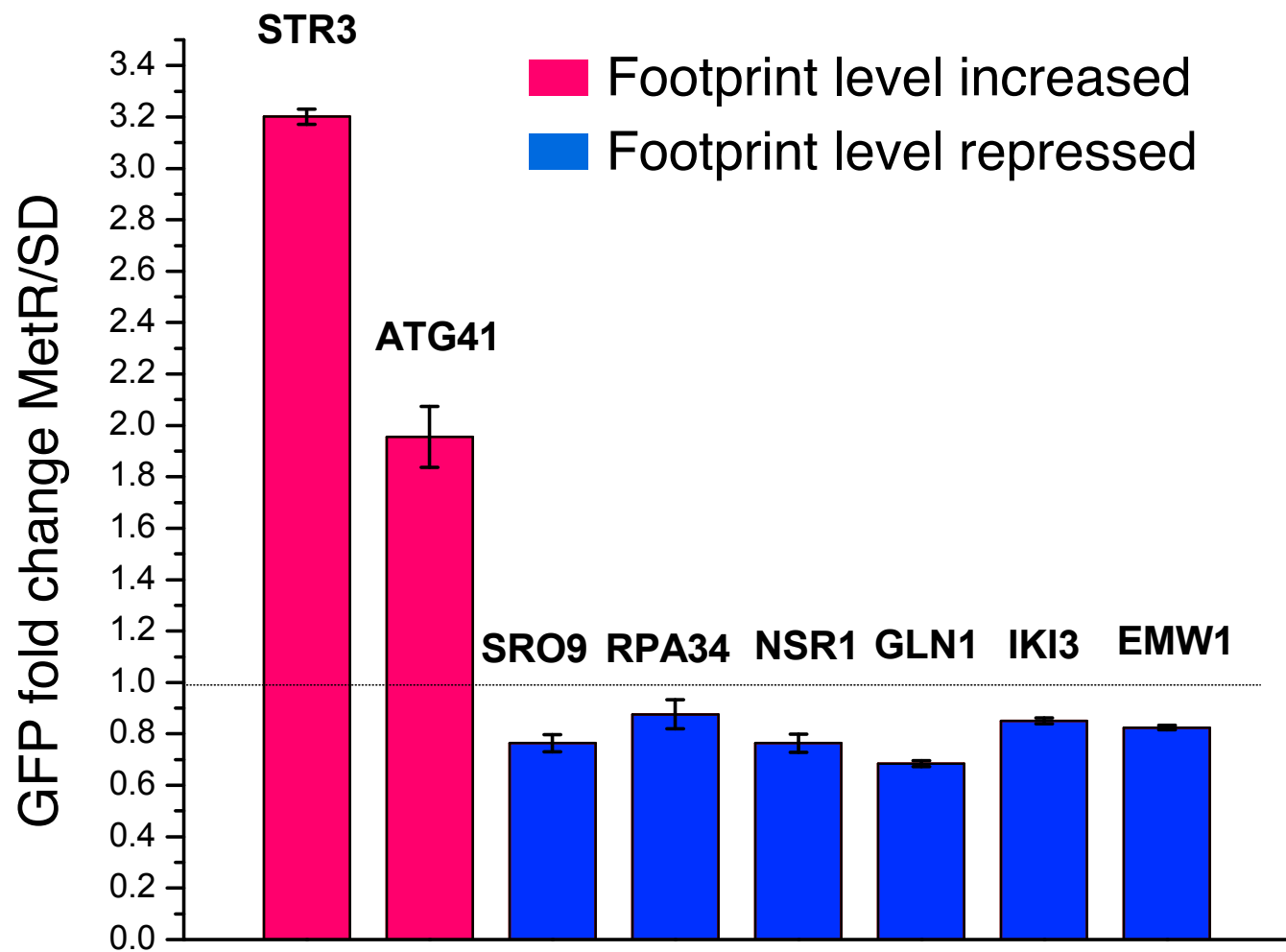

Supplement: Additional file 5: Figure S2. — Validation of protein level changes under MetR by flow cytometer. Red bars are genes with increased footprint reads while blue bars represent genes with decreased footprint reads. The mean GFP fold changes and error bars are computed from three biological replicates. (PDF 160 kb) [file 12864_2017_3483_MOESM5_ESM.pdf]

A

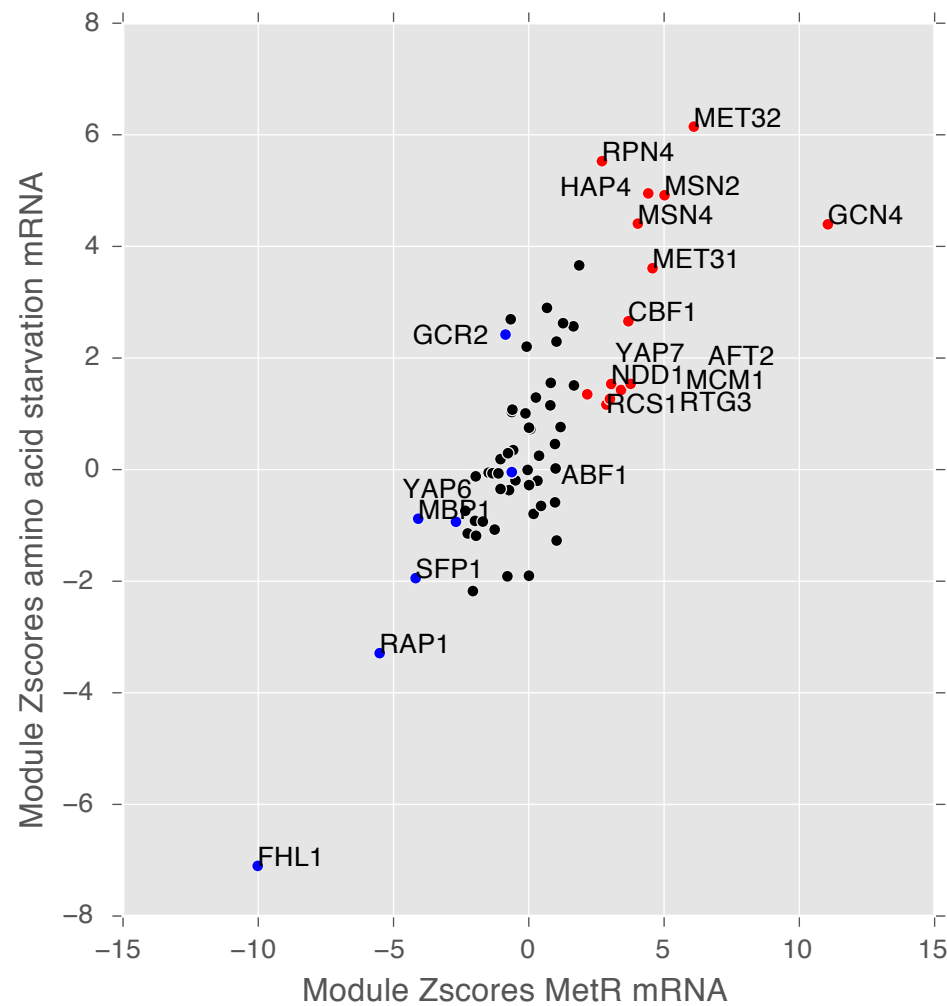

B

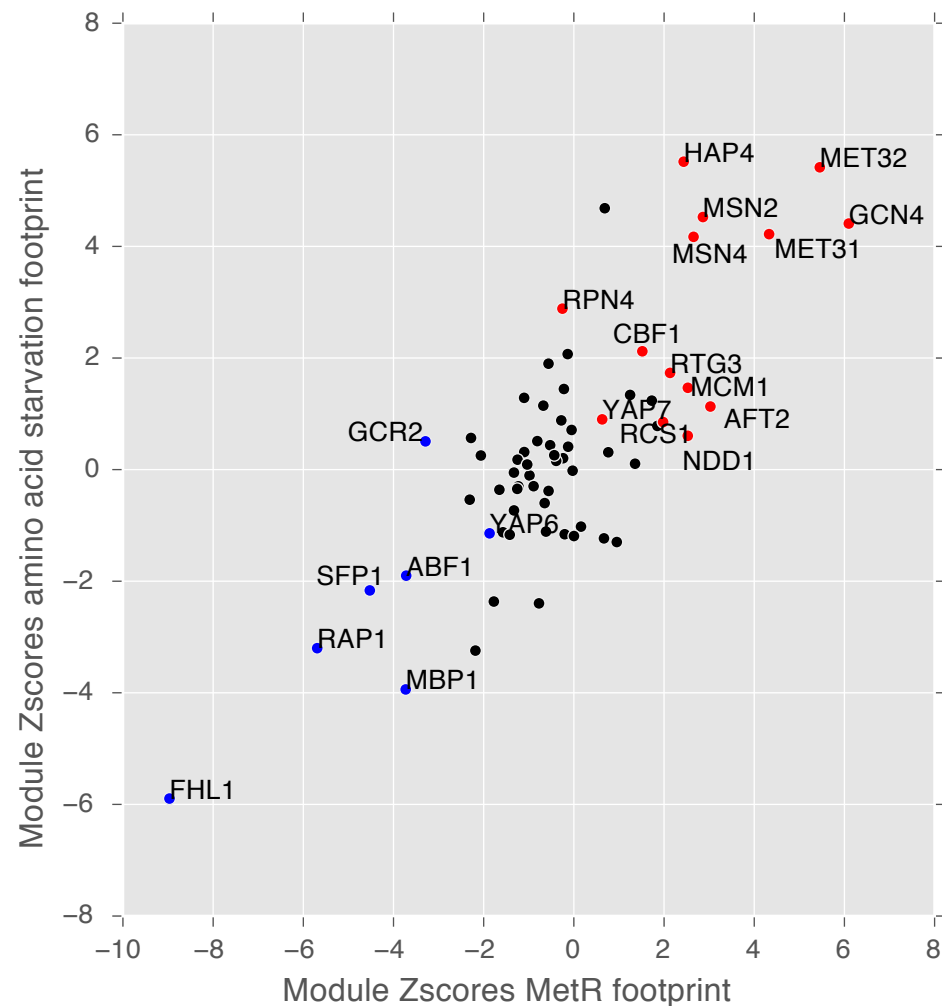

Supplement: Additional file 8: Figure S3. — Similar transcriptional and translational regulation under MetR and general amino acid starvation revealed by transcription factor modules: (A) module scores from mRNA data. (B) module scores from footprint data. (PDF 193 kb) [file 12864_2017_3483_MOESM8_ESM.pdf]
